# Washing and Squeezing Amalgams

**Published:** 1876-12

**Authors:** 


### ARTICLE VII.

### *Washing and Squeezing Amalgams.*

Very much has been said by *good authority* against both of these practices.

When mercury is mixed with the alloy, oxidation of the latter takes place to a certain extent, and if the black oxide is not removed by washing, it of course remains in the plug. This will give the tooth a darker appearance, *through the dentos*, than it would otherwise have. The exposed *surface* of the plug would also be more likely to turn dark.

Now, what is the *objection* to washing? It is *said* that the plug will be more likely to leak; to contract more at the surface on hardening. Also, that it is *difficult* to dry the amalgam, if washed. Well, the *truth* is easily found out in regard to this. Let every practitioner experiment with the amalgam which he uses, with glass test tubes. *Squeezing.* Any one will find on experiment that *more mercury* will remain in the amalgam after squeezing than would have been necessary, if the smallest *workable* quantity is sufficient.

For instance, take Hardman's Alloy, and follow his direction. Add mercury enough to make a good paste, after squeezing I found 80 of mercury to 100 of alloy.

Now, this same alloy, if weighed, requires just half its weight of mercury to make a workable amalgam—100 powder to 50 mercury. The more mercury used, the more oxidation is produced at the time of amalgamation, and, of course, the more black oxide will be washed away, if washed.

I have washed with bi-carbonate of soda several amalgams, finishing the washing with clear water, and thoroughly drying the mass by spreading and manipulating it on unglazed paper. These were then packed in glass tubes for leakage test, and none of them showed any signs of leakage after 24 hours immersion in aniline red.

Some amalgams will be very much altered if squeezed Gold, which is very soluble in mercury, will be squeezed out, and the amalgam deteriorated, probably. And so of tin also. Can any one give a good reason for *squeezing* amalgam ?

To get the best results, one ought to know just what he is using every time—*Missouri Dental Journal.*

---

### ARTICLE VIII.

### *Epithelioma of the Face.*

